# Network-based association analysis to infer new disease-gene relationships using large-scale protein interactions

**DOI:** 10.1371/journal.pone.0199435

**Published:** 2018-06-27

**Authors:** Apichat Suratanee, Kitiporn Plaimas

**Affiliations:** 1 Department of Mathematics, Faculty of Applied Science, King Mongkut’s University of Technology North Bangkok, Bangkok, Thailand; 2 Advanced Virtual and Intelligent Computing (AVIC) Center, Department of Mathematics and Computer Science, Faculty of Science, Chulalongkorn University, Bangkok, Thailand; National Center for Toxicological Research, UNITED STATES

## Abstract

Protein-protein interactions integrated with disease-gene associations represent important information for revealing protein functions under disease conditions to improve the prevention, diagnosis, and treatment of complex diseases. Although several studies have attempted to identify disease-gene associations, the number of possible disease-gene associations is very small. High-throughput technologies have been established experimentally to identify the association between genes and diseases. However, these techniques are still quite expensive, time consuming, and even difficult to perform. Thus, based on currently available data and knowledge, computational methods have served as alternatives to provide more possible associations to increase our understanding of disease mechanisms. Here, a new network-based algorithm, namely, Disease-Gene Association (DGA), was developed to calculate the association score of a query gene to a new possible set of diseases. First, a large-scale protein interaction network was constructed, and the relationship between two interacting proteins was calculated with regard to the disease relationship. Novel plausible disease-gene pairs were identified and statistically scored by our algorithm using neighboring protein information. The results yielded high performance for disease-gene prediction, with an F-measure of 0.78 and an AUC of 0.86. To identify promising candidates of disease-gene associations, the association coverage of genes and diseases were calculated and used with the association score to perform gene and disease selection. Based on gene selection, we identified promising pairs that exhibited evidence related to several important diseases, e.g., inflammation, lipid metabolism, inborn errors, xanthomatosis, cerebellar ataxia, cognitive deterioration, malignant neoplasms of the skin and malignant tumors of the cervix. Focusing on disease selection, we identified target genes that were important to blistering skin diseases and muscular dystrophy. In summary, our developed algorithm is simple, efficiently identifies disease–gene associations in the protein-protein interaction network and provides additional knowledge regarding disease-gene associations. This method can be generalized to other association studies to further advance biomedical science.

## Introduction

In cellular systems, proteins cooperate in various ways to accomplish needed functions. Therefore, dysfunction of proteins in multiple biological systems, such as DNA repair, apoptosis and immune functions, causes complex diseases [1, 2]. This complex nature has been studied extensively, but the exact character of associations between diseases and genes remains unclear. Thus, identification of genes associated with diseases is a challenging task in human genetics. It can help to reveal the molecular mechanisms of disease development, diagnosis and therapy. Several experimental methods have been established to identify disease-gene associations, such as genome-wide association studies (GWAS) [3], RNA interference (RNAi) screens [4], and linkage studies [5]. Since these methods are expensive and time consuming, many databases of disease genes have been developed, and computational methods have become an important tool to retrieve and analyze the disease data for a better understanding of disease mechanisms. Among the most commonly used databases of disease genes, Online Mendelian Inheritance in Man (OMIM) [6] and GeneCards [7] collect many manually curated data for the relationship between diseases and genes. Such relationships are inferred using data from gene variants [8, 9], biological pathways [10], gene expression data [11], biomedical ontologies [12] or text mining [13]. With this information, many studies have attempted to develop disease networks in which two connected diseases may have one or more shared genes, proteins, or microRNAs [14, 15]. Thus, these disease networks have become an important resource for analyzing the connections of genes and diseases. Not only disease networks but also gene or protein networks are useful to identify disease-gene associations [14, 16–20]. Many useful gene or protein networks have become widely used, such as the STRING database [21] for curated protein-protein interaction networks.

Karni et al. integrated a protein-protein interaction network with gene expression data under various disease conditions to predict causal genes [18]. They applied a greedy heuristic algorithm to identify a small set of disease-related genes that best explained the expression changes in disease-related genes with regard to pathways leading from causal to affected genes in the protein-protein interaction (PPI) network. Then, they predicted possible genes involved in myasthenia gravis. Goh et al. constructed a human disease network containing links between known genetic disorders and their corresponding disease genes in the human genome. Novel cancer-related genes were identified in their study [14]. Lee et al. constructed a bipartite human disease association network [19]. In their network, any two diseases were connected if their mutated enzymes associated with diseases catalyzed adjacent metabolic reactions. Their predicted disease associations were frequently identified in patients. In addition, patients who were diagnosed with a hub disease in the disease network were likely to develop other diseases connected to it regardless of previous diagnoses. Janjic and Przulj isolated a topologically and functionally homogeneous core subnetwork of a human PPI network and demonstrated that the subnetwork was enriched in disease genes and drug targets [20]. They hypothesized that wiring the core subnetwork can lead to disease development. The results of their study showed that new related diseases could be inferred by using the topology of their constructed network. After that, they identified modules or groups of disease-related genes. Notice that, although several studies have attempted to predict disease-gene associations, the number of possible disease-gene associations is very small [22].

To identify more disease-gene association, we developed an algorithm called “Disease-Gene Association (DGA)” based on *k* nearest neighbors and local network analysis of the large-scale human protein-protein interaction data integrated with disease relationships. We demonstrated the capability of inferring new diseases from existing known diseases when they share some functional information. In addition, we utilized information of disease-gene associations as a hint to infer new associations. As a new set of related diseases is typically difficult to identify, our tool attempts to identify some traces or hints from *k* neighboring genes of a query gene. Under the assumption that two interacting proteins share similar functions and play important roles in the same diseases, if a gene is associated with more than one disease, its interacting gene may also be related to those diseases in its module. A weighted protein-protein interaction network was first constructed and the disease relationship between two proteins in the network was determined with regard to disease associations by using various standard association indices. After that, association scores of a query gene and each of related diseases were calculated and used to filtering the best association candidates. Tenfold cross-validation was performed to evaluate the overall performance of disease-gene associations, and the validity of the scores was compared with the gold standard set. In addition, the performance of our method was compared with the performance of a random experiment, and the robustness of our method was investigated.

## Materials and methods

### Data sources of disease-gene associations and protein-protein interactions

**Fig 1:**
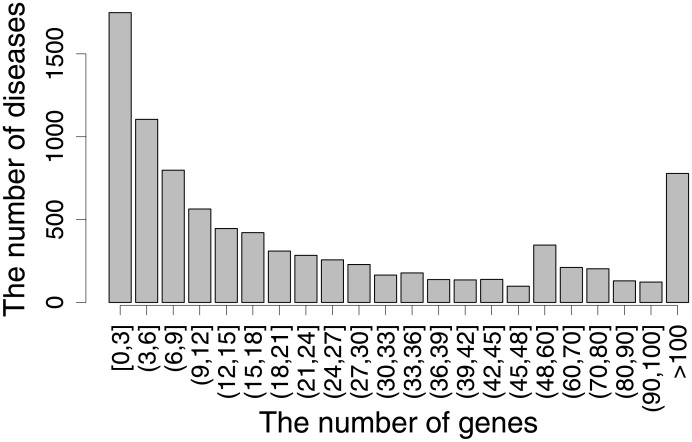
The relationship between the number of genes and diseases. The histogram illustrates the number of genes and their related diseases.

To infer new gene-disease associations, the gold standard of the gene and disease annotations were retrieved from DisGeNET (http://www.disgenet.org) [23]. DisGeNET is one of the largest available repositories of genes and variants involved in human diseases, including Mendelian, complex, environmental and rare diseases and disease-related traits. This database integrates data from expert curated repositories with information gathered through text-mining of the scientific literature, GWAS catalogues and animal models. With these data, we obtained a total of 15,081 diseases and 17,359 genes. We investigated the number of genes associated with diseases and found that numerous diseases were associated with a few known genes (see Fig 1, the histogram of the number of known gene-disease associations). Therefore, it is important to reveal new disease-gene associations to gain more knowledge of disease and gene mechanisms.

For reconstructing a protein-protein interaction network, we employed the human protein-protein interaction data retrieved from the STRING database version 10.5 [21]. This database contains known and predicted protein-protein interactions for both physical and functional interactions with confidence scores. The inversion of confidence scores were used as weights of the interactions in the network. To obtain only reliable interactions, we reconstructed the human interaction network by selecting only interactions with high confidence scores of greater than 900. This resulted in a constructed network containing 10,438 proteins and 250,312 interactions with their confidence scores to perform a *k*-nearest neighbor search in our algorithm. Gene symbols in this study were consistent with the standards described by the HUGO Gene Nomenclature Committee (www.genenames.org). Only genes with at least one known associated disease were considered the gold standard. In total, we obtained 13,291 diseases and 8,726 genes for our analysis to compute their disease-gene association score.

### Schematic overview of identifying disease-gene associations

**Fig 2:**
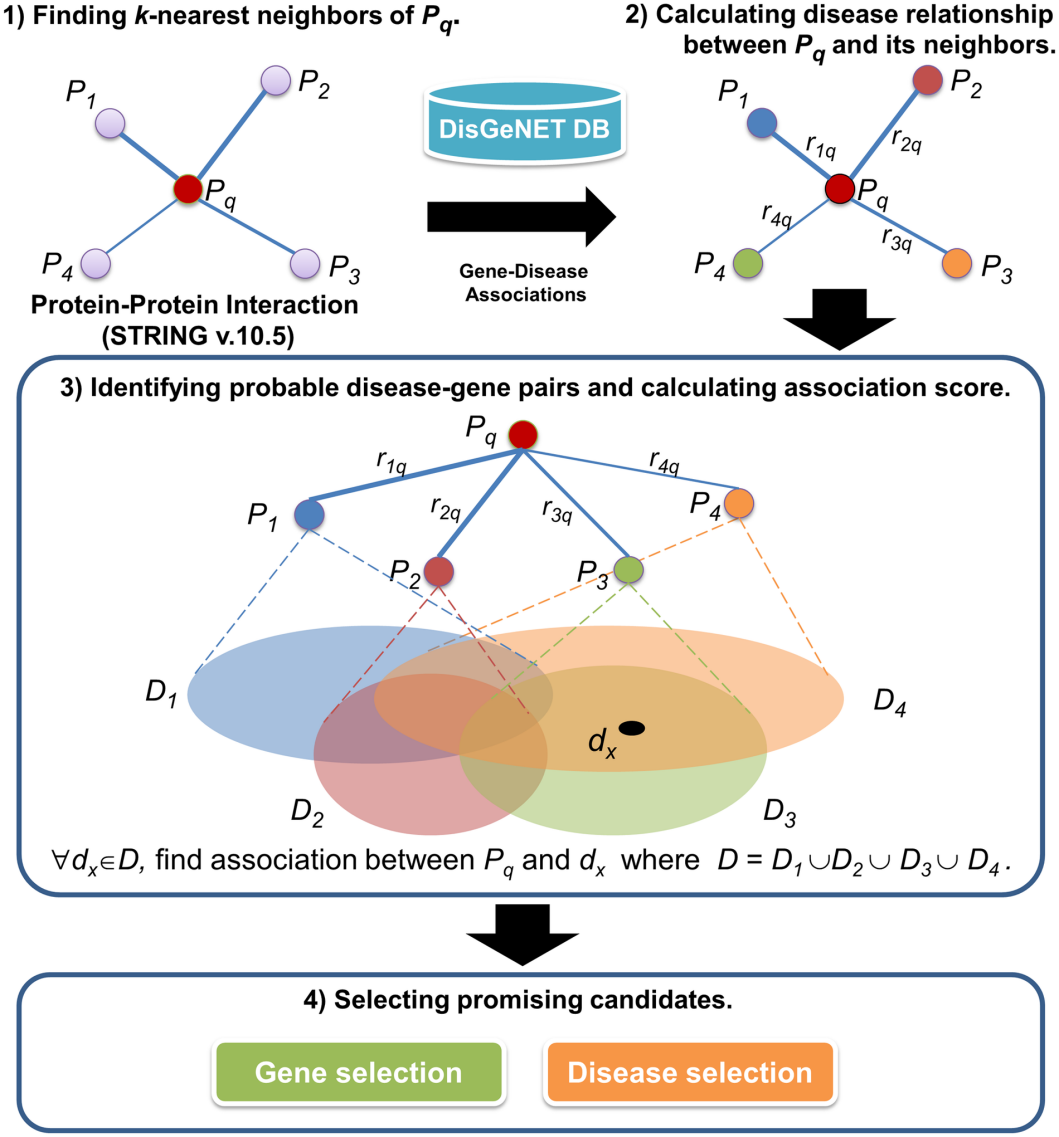
Schematic overview of identifying the disease-gene associations. Neighboring proteins of a query protein are sought using the inversion score from the STRING database, and their related diseases from DisGeNET are mapped. The disease relationship between two proteins is calculated using an association index. Then, the association scores of probable disease-gene pairs are calculated. Promising candidates were identified using gene and disease selections.

The general framework of our method is depicted in Fig 2. First, we constructed a protein-protein interaction network. The network was weighted using the inversion score from the STRING database [21]. With this network, nearest neighboring proteins of a query protein were sought using the network weights. The relationship between two proteins with regard to their disease relationship was calculated by an association index representing the relationship between two proteins in term of disease involvement.

The disease-gene data used for calculating the index were obtained from DisGeNET. The disease relationship was computed for all connected proteins in the network. This weighted network and the disease relationship were used to infer associations between a protein corresponding to a query gene and the set of related diseases that were obtained from neighbors of the query proteins. Suppose a query protein, namely, *P*_*q*_, has four nearest neighbor proteins, namely, *P*_*1*_, *P*_*2*_, *P*_*3*_ and *P*_*4*_, with the disease relationships between the query protein and its neighbors represented as *r*_*1q*_, *r*_*2q*_, *r*_*3q*_ and *r*_*4q*_, respectively. The sets of related diseases of the neighboring proteins were queried from the disease-gene database. We defined *D*_*i*_ as a set of related diseases for a protein *P*_*i*_. The union set of all related diseases is defined as a set *D* where *D* = {*D*_*1*_∪*D*_*2*_∪*D*_*3*_∪*D*_*4*_}. For all *d*_*x*_, where *d*_*x*_∈*D*, we identify the associations between *P*_*q*_ and *d*_*x*_ using our DGA algorithm. The association scores between all probable pairs of genes and diseases are calculated. Finally, promising candidates of disease-gene pairs are selected by considering gene or disease selections.

### Disease-gene association (DGA) algorithm for identifying probable disease-gene pairs and their association scores

To identify new associations between a gene and other diseases, we could not validate or perform predictions for all combinations of all available genes and diseases. Therefore, we required some hints to select possible diseases for investigating the relationship to a query gene. With this need, we found candidate diseases of a query gene by considering a set of diseases associated with *k*-nearest neighboring proteins of a query protein encoded by the query gene. The parameter *k* represented the number of nearest neighbor proteins of a query protein. To obtain the set of *k*-nearest neighboring proteins, we sought the nearest neighbors of the query protein based on an inversion of the interaction score of the PPI from the STRING database. With this finding, probable disease-gene pairs were obtained by considering all pairs between the query gene and the set of probable diseases. Then, the strength of the relationship of the disease-gene pairs was calculated and represented as a disease-gene association score. The disease-gene association (DGA) algorithm was implemented to calculate the association scores. The pseudocode of the DGA algorithm is illustrated in Algorithm 1, and a flowchart of this algorithm is illustrated in S1 Fig. In general, the PPI network can be modeled as a monopartite graph comprising only one type of node. The importance of the relationship between two proteins can vary depending on the studied problem. The relevance is typically represented as a value shared by two proteins. Suppose we have a weighted PPI network that indicates the relevance of two proteins with regards to the disease relationship. All candidate diseases of the query gene can be integrated as a universal set of diseases *D*. The association for each member *d*_*x*_ in *D* to the query gene can be validated by examining whether the member is included in the set of associated diseases of each neighboring protein. If the member is included, the disease relationship between the neighboring protein and the query protein can be aggregated. We performed this process for all *k*-nearest neighbor proteins and obtained the associated score between the query gene and the disease *d*_*x*_. The final score was calculated by the aggregated association value divided by the number of neighboring proteins of the query protein and multiplied by 100. Therefore, the range of the association score was between 0 and 100.

### Algorithm 1: Disease-Gene Association (DGA)

Input: 1) Weighted_network (a PPI network weighted by inversion of interaction score from STRING)

  2) A set of known diseases associated to gi (Dgi) for all genes in the network.

Output: Disease-gene_association_score

**1**. **for each** gene *g*_*i*_ in Weighted_network **do**

**2**.  Find *NN*_*genes* (*k* nearest neighboring genes of *g*_*i*_)

**3**.  $D=\cup_{nn=1}^{k}D_{g_{nn}}$

**4**.  **for each** *d*_*x*_ **in** *D* **do**

**5**.    *score* ≔ 0

**6**.    **for each** gene *g*_*nn*_ in *NN_genes* of *g*_*i*_ **do**

**7**.     **if** *d*_*x*_ **∈** *Dg*_*nn*_ **then**

**8**.      *score* ≔ *score* + *disease*_*relationship*(*g*_*i*_, *g*_*nn*_)

**9**.     **endif**

**10**.    **endfor**

**11**.   Disease-gene_association_score(*g*_*i*_, *d*_*x*_) ≔ (100*score)/*k*

**12**.  **endfor**

**13**. **endfor**

### Association indices

To calculate the disease relationship of an interaction in the PPI network, we considered the relationship in terms of the two genes involved in the same set of diseases. To identify such a relationship, we employed the disease-gene data from the gold standard that provided connections between a disease and its known related genes. Genes associated with a disease were mapped to their products in the PPI network. A measurement that could be employed to identify the disease relationship between two proteins on the network is an association index. The association index was applied to calculate the relevance between two corresponding proteins of the genes. Well-known association indices include the Jaccard index, Simpson index, Geometric index and Cosine index. These indices are used for measuring profile similarity between two proteins on a graph. The similarity between two interacting proteins is determined based on the number of shared disease nodes and the total number of diseases connected to these gene products. Each type of index measures the sharing via different methods. The Jaccard index measures the proportion of shared disease nodes between two genes relative to the total number of disease nodes connected to the two genes. The Simpson index is similar to the Jaccard index. The Simpson index considers the proportion of shared disease nodes relative to the degree of the least connected node. The Geometric index calculates the product of the proportion of shared nodes between two genes. The Cosine index calculates the similarity of shared nodes between two genes via a geometric method.

For example, if we define *Dg*_*1*_ as the set of diseases associated with gene *g*_*1*_ and *Dg*_*2*_ as the set of diseases associated with gene *g*_*2*_. | *Dg*_*1*_ | and | *Dg*_*2*_ | are the number of diseases associated with *g*_*1*_ and *g*_*2*_, respectively. | *Dg*_*1*_ ∩ *Dg*_*2*_ | indicates the number of shared partners of *Dg*_*1*_ and *Dg*_*2*_. The calculations of these indices are summarized in Table 1.

**Table 1:** Summarization of standard association indices for calculating disease relationships.

Association index	Formula
Jaccard index	$\text{Jaccard}\left(g_{1},\;g_{2}\right)=\frac{\left|Dg_{1}\cap Dg_{2}\right|}{\left|Dg_{1}\cup Dg_{2}\right|}$
Simpson index	$\text{Simpson}\left(g_{1},\;g_{2}\right)=\frac{\left|Dg_{1}\cap Dg_{2}\right|}{\text{min}\left(\left|Dg_{1}\right|,\left|Dg_{2}\right|\right)}$
Geometric index	$\text{Geometric}\left(g_{1},\;g_{2}\right)=\frac{{\left|Dg_{1}\cap Dg_{2}\right|}^{2}}{\left|Dg_{1}\right|\cdot \left|Dg_{2}\right|}$
Cosine index	$\text{Cosine}\left(g_{1},\;g_{2}\right)=\frac{\left|Dg_{1}\cap Dg_{2}\right|}{\sqrt{\left|Dg_{1}\right|\cdot \left|Dg_{2}\right|}}$

### Performance measurement

To determine the performance of our DGA algorithm, a tenfold cross-validation approach was employed to evaluate the accuracy of the predictions as follows. First, the order of disease-gene pairs was randomized. After that, these randomly ordered disease-gene pairs were partitioned into ten parts, each consisting of approximately 10% of the disease-gene pairs. We then iterated over those parts, where at each iteration we hid the disease-gene connections that were included in the current part and used the remaining pairs to calculate disease relationship for each protein interaction by any association indices. Notice that for each iteration during the cross-validation procedure, the association indices for each protein interaction were re-computed based on the remaining disease-gene pairs. Then, disease-gene association scores were computed by our DGA algorithm for the held-out disease-gene pairs. With the tenfold cross-validation, all of the disease-gene pairs were given association scores to compare to the gold standard list. ROC curves and the F-measure were used to measure the prediction performance of this scenario. Furthermore, this scenario was performed three times to obtain a list of predictions for performance evaluation.

In general, the number of known associations is much smaller than the number of all protein-disease pairs. To avoid bias from highly imbalanced data between these two sets, we performed a bootstrap resampling technique by selecting an equal number of data between these two groups and measuring the performance. This process was repeated five times, and the overall performances were calculated by the mean value of these performances. To calculate the performance, precision and recall (sensitivity) were calculated in our predictions. For a query gene, the association scores between a gene and related diseases of its neighboring proteins coded by the query gene were investigated. Notice that some of these related diseases may be new or may have been previously known for the query gene. Using our gold standard of disease-gene associations, a positive set was determined. Therefore, the true positive set was the set of predicted associations that were found in the gold standard. The false positive set was the set of predicted associations that were not found in the gold standard, and the false negative set was the set of disease-gene pairs that were not predicted to be associated but were found in the gold standard. With these sets, recall and precision were calculated. Given a certain threshold of the association scores, precision is the ratio of true positive disease-gene pairs whose association scores are above the threshold to the total number of disease-gene pairs whose association scores are above the threshold (so-called predicted list under a certain scoring threshold). Recall is the ratio of true positive disease-gene pairs whose association scores are above the threshold to the total number of true disease-gene pairs in the dataset. From these measures, the F-measure can be calculated as follows:

$$F-measure=2*\frac{precision*recall}{precision+recall}.$$

The F-measure evaluates the overall effectiveness of the classification. Higher F-measure values indicate better overall performance in terms of emphasizing a positive class. While the use of the F-measure represents the prediction performance only for a certain threshold, ROC curves can be used to measure the overall performance over a range of the association scores. For any certain score, the true positive rate and false positive rate are calculated and plotted on a curve. The ROC curve shows how much better the scores are for predictions compared with a random selection. If the curve is a diagonal line, it was a random prediction. Thus, the area under the curve was 0.5. If the curve is not a diagonal line and has an area under the curve more than 0.5, this shows a better performance than a random prediction.

### Association coverage of a gene and a disease

With a large number of prediction results, we focused on some specific diseases or genes that exhibited promising results. The predicted disease-gene associations with association scores can be verified if they were previously known in the gold standard. In general, a gene can be associated with more than one disease, and a disease is also associated with multiple genes. To identify diseases that were relevant to certain genes, we focused on a disease for which the majority of the predicted associations of that disease and gene were known in our gold standard. We defined such a disease to have a high association coverage value for that disease Therefore, the coverage value was calculated as the number of predicted disease-gene pairs of a disease found in the gold standard divided by the number of all predicted disease-gene pairs of that disease. In the same manner, we identified genes with high association coverage by calculating the number of predicted disease-gene pairs of the gene that was identified in the gold standard divided by the number of all predicted disease-gene pairs of that gene.

## Results

### DGA algorithm with Jaccard index yields the best performance to identify disease-gene associations

**Fig 3:**
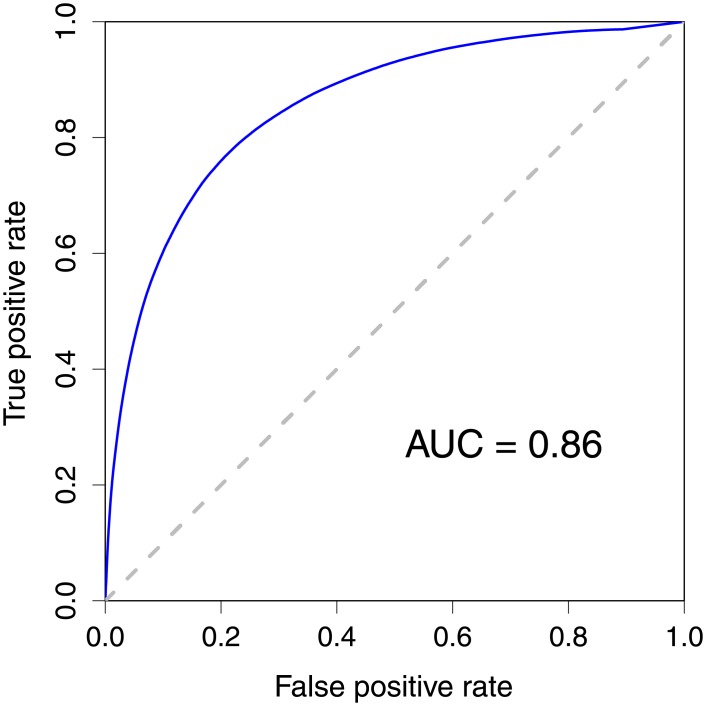
Receiver operating characteristic curves for the predictions of disease-gene association. The disease-gene prediction results at the optimal parameter *k* = 18.

The DGA algorithm is a generalized method that was performed on the weighted PPI network with adaptable relationship between proteins. We started our analysis using the weighted PPI network in which the disease relationship between two proteins was calculated using the Jaccard index. The DGA algorithm investigated the neighboring proteins of a protein coded by a query gene. Therefore, the number of neighbors (*k*) is a parameter that can be optimized. We varied the parameter *k* from 1 to 30. The F-measure was calculated to measure the performance of the DGA algorithm for each parameter *k* with different score cut-off thresholds. We performed the tenfold cross-validation to evaluate our method. We used the highest F-measure values of all F-measure values from different thresholds as the F-measure value for each parameter *k*. The algorithm yielded good results with high performance. Investigating the F-measure values of the DGA with the PPI network using the Jaccard index value as a disease relationship, F-measure values between 0.75 and 0.78 were obtained for all *k* values. The F-measure value increased gradually between the smallest *k* values (*k* = 1) and the parameter *k* = 18, and then decreased slightly. Therefore, we selected the smallest *k* value (*k* = 18) as our optimal parameter *k*, which yielded the area under the receiver operating characteristic (ROC) curve of 0.86 (See Fig 3) and the highest F-measure of 0.78, and 508,717 true positives, 152,215 false positives, 128,552 false negatives, and 484,053 true negatives for balanced data. The complete list of 3,789,655 disease-gene pairs with association scores is presented in S1 Table.

**Fig 4:**
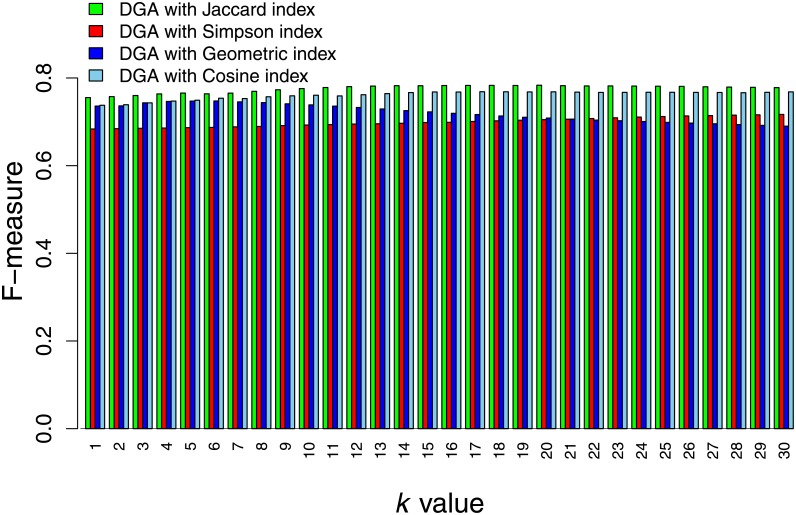
DGA algorithm performance. The highest F-measure from cross-validation results from the DGA algorithm with different disease relationships calculated using the Jaccard, Simpson, Geometric, and Cosine indices.

We also investigated disease relationships calculated by other association indices and attempted to select the optimal index that yielded the best performance. The investigated association indices should provide association values between 0 and 1. In this study, we considered the Simpson, Geometric, and Cosine indices (see “Materials and methods”). The DGA algorithm was performed on the weighted network with the same gold standard. The results showed that DGA with disease relationships from the Jaccard index value yielded superior performance. The performance was slightly lower for the DGA with disease relationships from Geometric index values at small *k* values, and the remainders of the F-measure values were similar when the *k* value increased. The third-ranked algorithm was the DGA with disease relationships from the Cosine index. This index exhibited slightly different performances compared with the DGA with disease relationships from the Jaccard and Geometric indices at small *k* values. The worst performing algorithm was the DGA with disease relationships from the Simpson index. Its performance was considerably reduced compared with other indices for all *k* values. Based on these results, we selected the DGA with disease relationships from the Jaccard index for our predictions. A comparison of the index performances is presented in Fig 4.

### Disease relationships provide a good support to the DGA algorithm

**Fig 5:**
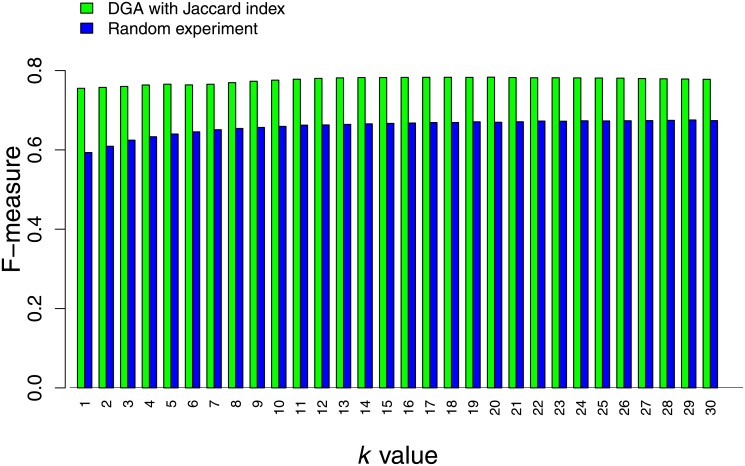
Comparing performance of our method and random experiment. The disease-gene association predictions using DGA algorithm with disease relationships from the Jaccard index and random experiments with different values of *k* were compared.

To demonstrate the effectiveness of using disease relationship in the DGA algorithm, we examined our algorithm by randomly shuffling the values of disease relationships. Then, the DGA algorithm was performed to calculate association scores for each disease-gene pairs. This scenario was repeated 3 times for all *k* values. As expected, we obtained very low average F-measure values for all *k* values. For each value of *k*, we observed a decreasing tendency of F-measure values. The F-measure values were higher for the smallest values of the association score threshold and were reduced to values closer to zero when the threshold increased to the maximum threshold. The best average F-measure value obtained from these random experiments was 0.67, while the performance of our algorithm with the true disease relationship gave the F-measure of 0.78. The results indicate that the performance of our method is higher than the performance of the random experiments. Comparison of the performances of the true disease relationship and the random relationship is presented in Fig 5.

### Robustness of network interference

The DGA algorithm inferred protein-disease associations based on the structure of the interaction network. It was necessary to validate whether the algorithm was sensitive to the network structure. To perturb the network, the original network was altered by removing important nodes in the network. The important nodes were defined as high degree nodes. Therefore, we performed the experiments by removing nodes with greater than 300, 200, and 100 degrees and then used the altered network in our analysis framework and investigated the prediction performance. The first altered network was constructed by removing 126 proteins with greater than 300 node degrees. The performance was similar to the original network, and the altered network exhibited the same tendencies when the parameter *k* increased. The second and third networks were constructed by removing 596 and 1,716 proteins with greater than 200 and 100 node degrees, respectively. The performance of the algorithm on the second network was slightly reduced compared to the performance of the algorithm on the original and the first altered network. The F-measure values of the first and the second experiments ranged between 0.74 and 0.78 and were quite similar to the F-measure value from the original network. The same tendencies were noted for the algorithm in the third experiment. The results yielded slightly reduced F-measure values compared with the performance on the other networks. The F-measure values ranged between 0.74 and 0.76. These results revealed the robustness of the DGA algorithm on the altered network.

### Investigating our algorithm on a protein complex dataset

**Fig 6:**
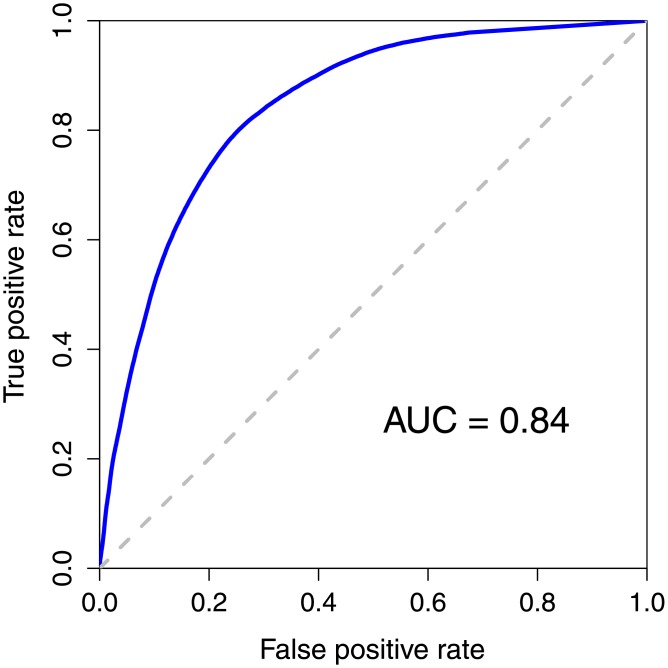
Receiver operating characteristic curves for the prediction of disease-gene associations using interactions from protein complexes. The disease-gene prediction results using our DGA algorithm with interactions from protein complexes obtained from the CORUM database.

Instead of employing interactions from the STRING database, we applied our DGA algorithm to interactions that were reported within the same complex in CORUM database [24]. Without defining nearest neighboring proteins in the same complex, all proteins that were in the same complex were defined as neighboring proteins of each other in our algorithm. Therefore, the parameter *k* was discarded. With the core complex data from CORUM and disease-gene database from DisGeNET, we obtain a total of 8,090 query proteins and 1,679,974 disease-gene pairs for our analysis. Applying these data to the DGA algorithm and evaluating the performance by tenfold cross-validation, we obtained the best F-measure of 0.74 and the AUC of 0.84. The ROC plot of our prediction with this data set is shown in Fig 6. The performance showed the same tendency as when we used interactions from STRING. With good interaction data from STRING or CORUM, our algorithm could mine disease-gene association accurately with good prediction performances.

### Promising disease-gene candidates based on gene selection with high coverage values

Using a large set of 3,789,654 predicted disease-gene associations of 8,723 genes from the prediction with the optimal parameter *k*, we performed a post-processing procedure that considered both the association score and coverage value to filter the predicted results to obtain promising candidates. Under these conditions, we focused on two issues. We selected genes that exhibit high coverage values or diseases that have high coverage values. Then, we used the association score of those selected genes or diseases to filter the results. Histograms of the association coverage of genes and diseases, and association scores are presented in S2, S3 and S4 Figs, respectively. The association coverage values of all 8,723 genes are presented in S2 Table. In addition, S3 Table presents the association coverage values of all 13,103 diseases from the results with optimal parameter *k* value. Focusing on gene selection, we used stringent criteria, with coverage value of a gene at 70 and an association score of 40 as our thresholds. Thus, only genes that have coverage values of genes greater than or equal to 70 and disease-gene pairs of these genes that have an association score greater than or equal to 40 were selected as our promising candidates. Based on these selection criteria, we obtained 134 disease-gene associations with 6 proteins/genes, including

1. late cornified envelope 3B (*LCE3B*),
2. late cornified envelope 3C (*LCE3C*),
3. queuine tRNA-ribosyltransferase catalytic subunit 1 (*QTRT1*),
4. SPG11, spatacsin vesicle trafficking associated (*SPG11*),
5. serpin family B member 3 (*SERPINB3*), and
6. ATP binding cassette subfamily G member 8 (*ABCG8*).

Table 2 presents the coverage values and association scores of these 6 genes. In this list, only *LCE3C* had a precision of 1, indicating that the prediction results of *LCE3C* associated with diseases were previously known in the gold standard. The interesting predicted results are the set of associations that were not currently known in the gold standard. These new associations are presented in Table 3. A list of all 134 predicted disease-gene associations that met the criteria of an association score greater than or equal to 40 and coverage value greater than or equal to 70 is presented in S4 Table.

**Table 2:** List of genes that met the selection criteria with coverage value of a gene greater than or equal to 70 and an association score greater than or equal to 40.

Gene Symbol	Coverage Value of a Gene
*LCE3C*	100.00
*LCE3B*	90.91
*QTRT1*	79.31
*SPG11*	77.78
*SERPINB3*	77.14
*ABCG8*	74.19

**Table 3:** List of predicted disease and gene pairs that were not evident in the gold standard.

Gene Symbol	Disease	Association Score
*LCE3B*	NEUROTICISM	90.91
*QTRT1*	Ataxia	67.65
*QTRT1*	Waldenstrom Macroglobulinemia	67.65
*QTRT1*	nervous system disorder	67.65
*QTRT1*	Neuroblastoma	67.65
*QTRT1*	Neuromuscular Diseases	67.65
*QTRT1*	Central neuroblastoma	67.65
*SERPINB3*	Malignant neoplasm of skin	49.09
*SERPINB3*	Malignant tumor of cervix	49.09
*SERPINB3*	Neoplasm Metastasis	49.09
*SERPINB3*	Skin Neoplasms	49.09
*SERPINB3*	Uterine Cancer	49.09
*SERPINB3*	Cervix carcinoma	49.09
*SERPINB3*	Squamous cell carcinoma of skin	49.09
*SERPINB3*	Carcinoma of larynx	49.09
*ABCG8*	Alzheimer’s Disease	46.00
*ABCG8*	Inflammation	46.00
*ABCG8*	Lipid Metabolism, Inborn Errors	46.00
*ABCG8*	melanoma	46.00
*ABCG8*	Prostatic Neoplasms	46.00
*ABCG8*	Xanthomatosis	46.00
*ABCG8*	stomatocytic anemia	46.00
*ABCG8*	Disorder of macula of retina	46.00
*SPG11*	Malignant neoplasm of breast	42.42
*SPG11*	Cerebellar Ataxia	42.42
*SPG11*	Breast Carcinoma	42.42
*SPG11*	Cognitive deterioration	42.42

Only associations that met the criteria of the coverage value of gene and association score and were not evident in the gold standard were selected for inclusion in the search for literature-based evidence. For the predicted list of 134 associations based on gene selection, we attempted to find literature-based evidence to support the predicted associations. Our method demonstrated that *SERPINB3* is associated with 8 new diseases as shown in Table 3. *SERPINB3* is a member of the serpin superfamily of protease inhibitors. *SERPINB3* is involved in apoptosis, immune responses, blood coagulation, cell migration and invasiveness of cells [25, 26]. *SERPINB3* is also known as squamous cell carcinoma antigen 1 (*SCCA1*). It was first identified in squamous cell carcinoma tissue from the cervix of women [27] but it is physiologically found in normal squamous epithelium [28, 29]. *SERPINB3* is highly expressed in tumors of epithelial origin, including hepatocellular carcinoma [30, 31]. In addition, upregulation of *SERPINB3* is associated with benign hyperplasia [32]. *SERPINB3* is highly expressed in psoriasis [33], cutaneous SCC [34], and all SCC specimens as well as psoriasis [32]. In our predictions, we found that *SERPINB3* is associated with malignant tumors of the cervix and malignant neoplasms of the skin. These results seem to be reasonable. We also predicted that *SERPINB3* was associated with uterine cancer and carcinoma of the larynx. However, we have not found evidence to support these predictions.

The prediction results also revealed new associations for *ABCG8*. *ABCG8* was predicted to be associated with Alzheimer’s disease, inflammation, lipid metabolism, inborn errors, melanoma, prostatic neoplasms, xanthomatosis, stomatocytic anemia, and macular of retina disorders. *ABCG8* is a member of the superfamily of ATP-binding cassette (*ABC*) transporters that play important roles in the regulation of cellular cholesterol homeostasis. The accumulation of excess cholesterol is thought to contribute to the early onset of Alzheimer’s disease [35]. Therefore, *ABCG8* may relate to Alzheimer’s disease. In addition, *ABC* transporters are also involved in blood pressure regulation, endothelial function, vascular inflammation, and platelet production and aggregation [36]. Several studies describe connections of *ABC* transporters, cholesterol, and inflammation in liver. The expression of *ABCG8*, a transporter involved in free cholesterol excretion, was significantly reduced in the area of inflammation mediated by zymosan, a yeast glucan, on multiple steps in the RCT pathway in vivo and ex vivo [37].

Moreover, homozygous or compound heterozygous mutations in either *ABCG5* or *ABCG8* were observed in patients with sitosterolemia, an inborn error of metabolism. Sitosterolemia is a rare autosomal recessive lipoprotein metabolic disorder [38, 39]. The mutations lead to a complete loss of function of ATP-binding cassette (*ABC*) heterodimer transporter G5-G8. The loss of function of the transporter *ABCG5* and *ABCG8* increases the concentrations of plasma plant sterols [40]. Patients with sitosterolemia also exhibit xanthomas [41–43]. Infant patients exhibit intertriginous xanthomas on the heels and elbows [38, 41–43].

Our results also revealed the associations between *SPG11* and four different diseases as shown in Table 3. *SPG11* mutations are associated with a severe and complex form of autosomal recessive hereditary spastic paraplegias (HSP) with thin corpus callosum [44]. Mutations in the *SPG11* gene are noted in approximately 60% of patients exhibiting cognitive impairment and thin corpus callosum [45]. HSP are a large group of neurodegenerative disorders that share some common clinical characteristics of lower limb spasticity and weakness. Manifestations of the various forms of HSP range from congenital brain abnormalities, e.g., agenesis of the corpus callosum or cerebellar dysplasia, to signs of neuronal dysfunction and neurodegeneration, e.g., cognitive impairment, ataxia, optic nerve atrophy, epilepsy, and peripheral neuropathy [46, 47].

### Promising disease-gene candidates based on disease selection

Focusing on disease selection, we used different criteria. Diseases with coverage values greater than 40 and disease-gene pairs of these diseases with association scores greater than 20 were selected. Under these conditions, we obtained 37 disease-gene pairs with 23 different diseases. S5 Table presents all 37 disease-gene pairs with coverage values of diseases and association scores. The results revealed that most of these pairs were previously known in the gold standard, with the exception of (i) keratin 10 (*KRT10*) and epidermolysis bullosa simplex with mottled pigmentation (EBS-MP) and (ii) protein O-mannosyltransferase 1 (*POMT1*) and muscle biopsy exhibiting dystrophic changes. Therefore, we investigated all predictions of both diseases. Epidermolysis bullosa simplex with mottled pigmentation was predicted to be associated with three genes: keratins 5, 10, and 14. *KRT5* and *KRT14* have previously been associated with this disease. Therefore, *KRT10* represents a new gene associated with this disease. Five genes were predicted to be associated with muscle biopsy exhibiting dystrophic changes: *POMT1*, protein O-mannosyltransferase 2 (*POMT2*), dystroglycan 1 (*DAG1*), fukutin (*FKTN*), and fukutin related protein (*FKRP*). *POMT2* and *FKRP* were previously known to be associated with this disease. Therefore, 3 new genes were identified for this disease.

EBS-MP is a rare subtype of epidermolysis bullosa simplex (EBS) presenting blistering, mottled pigmentation of the trunk and limbs; punctate hyperkeratosis of the palms and soles; and dystrophic nails[48]. EBS-MP is caused by mutations in *KRT5* and *KRT14* [49]. A mutation in the *KRT5* tail (V2) domain was identified in a Japanese family characterized with skin fragility and pigmentary changes reminiscent of EBS-MP [50]. A recurrent missense mutation of *KRT14* was identified in a young patient with clinical and pathological features typical of EBS-MP [51]. The *KRT10* gene encodes keratin 10, which is produced in keratinocytes in the outer layer of the skin. Thus, it is possible that KRT10 is associated with EBS-MP as demonstrated by our results. Muscle biopsy exhibiting dystrophic changes is a synonym for muscular dystrophy. This term describes primary myopathies with a genetic basis and a progressive course. The condition is characterized by destruction of muscle and its replacement by fatty and fibrous tissue [52]. Mutations in *POMT2* cause severe congenital muscular dystrophy and are associated with a milder limb-girdle muscular dystrophy phenotype [53]. Fukutin-related protein (*FKRP*) was identified based on its sequence homology with fukutin [54]. Mutations in the *FKRP* gene cause limb-girdle muscular dystrophy type 2I (*LGMD2I*), an autosomal recessive hereditary disorder [55]. Associations of *FKTN*, *POMT1*, and *DAG1* with the muscle biopsy exhibiting dystrophic changes were not identified in our gold standard. However, some studies demonstrate that *FKTN*, *POMT1*, and *DAG1* are involved in muscular dystrophy [56–59]. Therefore, these three genes may be important targets for further evaluation.

## Conclusions and discussion

Decoding links between genes and diseases provides the opportunity to understand disease etiology and improve drug design and therapy. In this study, we exploited data from protein-protein interactions and disease-gene relationships to discover new disease-gene associations. The main hypothesis of this work involves the potential relatedness of genes sharing the same set of diseases. The relationship between two genes was considered in terms of their disease involvement. Our developed algorithm revealed novel disease-gene associations using information from neighboring proteins. An association index was applied to identify the association between two genes regarding disease involvement. Our results revealed that the DGA algorithm with a disease relationship from the Jaccard index yielded the best results compared with other standard association indices.

We realized the issue that STRING database also contains many predicted interactions. Using predicted interactions to infer new disease-gene associations is less reliable than using curated interactions from known pathways. Thus, in our analysis, only interactions with high confidence score were selected to ensure that the constructed protein-protein interaction network was reliable enough to be used to infer new connections or links among proteins. In addition, the interacting proteins need to be related to at least one disease to retrieve curated disease genes or proteins. With these criteria, we found that all of the analyzed interactions were evidenced in the database channel of STRING database in which the curated interactions were extracted from pathway databases such as KEGG, Biocarta, BioCyc, Gene Ontology, and Reactome. Therefore, the analyzed protein interaction network in this study was constructed and curated in the way to ensure that it contains mostly reliable interactions to infer new disease-gene associations.

We found that our algorithm produced low association score and coverage values either for genes or diseases. Based on the distributions of these values and statistical values, we found that most of scores and values were near zero. Mean association score and association coverage of genes and diseases values were 1.19, 6.01, and 1.94, respectively. This situation could have occurred due to the small number of known disease-gene associations. As demonstrated in the Materials and Methods section, our algorithm calculated the relationship in terms of disease involvement. In cases wherein two genes were not associated with each other, it is reasonable that the association score was zero. However, in cases where two genes were related but most of their associations with the same disease mechanisms have not been revealed to date, we also obtained a low association score. Thus, we need to increase the number of disease-gene associations. Our findings help to reveal some information about diseases and genes and fulfill some lacking associations regarding disease mechanisms. Based on our criteria for selecting candidates for biological interpretation, score values of 20, 40, and 70 were employed. These values were not low based on the distributions of association scores and coverage values. In addition, we could change any criteria that we preferred and choose new associations as candidates for further investigations in the laboratory. A limitation of our algorithm is that we analyzed only genes known to be related to at least one disease. The sets of known diseases were required because the disease relationship between two interacting genes calculated by an association index counted the number of shared related diseases.

The developed DGA algorithm is a generalized method when using different network data sets. As demonstrated in the Materials and Methods, the relevance between two proteins can be adaptable depending on the studied problem. One example of this adaptation was shown in the Results section, where we used interactions from the protein complex in the DGA algorithm and obtained a good performance. In this study, we were interested in the relationship between two proteins with regard to disease involvement. However, we could change the type of relationship that we applied the DGA algorithm to according to the nature of our research interest. In addition, instead of applying the algorithm to protein-protein interactions, we could apply the algorithm to any network of interest.

In conclusion, our developed method is simple and effective for exploring new disease-gene associations. Obtaining promising associations increases the possibility of gaining more knowledge to understand more functions of proteins in disease mechanisms, thereby increasing the opportunity for successful experimental solutions in biomedical studies.

## Supporting information

S1 Table List of all 3,789,655 disease-gene pairs with association scores. (ZIP)

S2 Table The coverage values of all 8,723 genes. (XLS)

S3 Table The coverage values of all 13,103 diseases. (XLS)

S4 Table List of all 134 predicted disease-gene associations that met the criteria of an association score greater than or equal to 40 and coverage value of a gene greater than or equal to 70. (XLS)

S5 Table List of all 37 predicted disease-gene associations that met the criteria of an association score greater than 20 and coverage value of a disease greater than 40. (XLS)

S1 Fig Flowchart of the DGA algorithm. (TIF)

S2 Fig Histogram of the coverage values of genes. (EPS)

S3 Fig Histogram of the coverage values of diseases. (EPS)

S4 Fig Histogram of the association scores. (EPS)
