# Supplementary figures and images for: Network-based association analysis to infer new disease-gene relationships using large-scale protein interactions
Source: PLoS One. 2018 Jun 27;13(6):e0199435. doi: 10.1371/journal.pone.0199435 (PMC6021074; doi:10.1371/journal.pone.0199435)

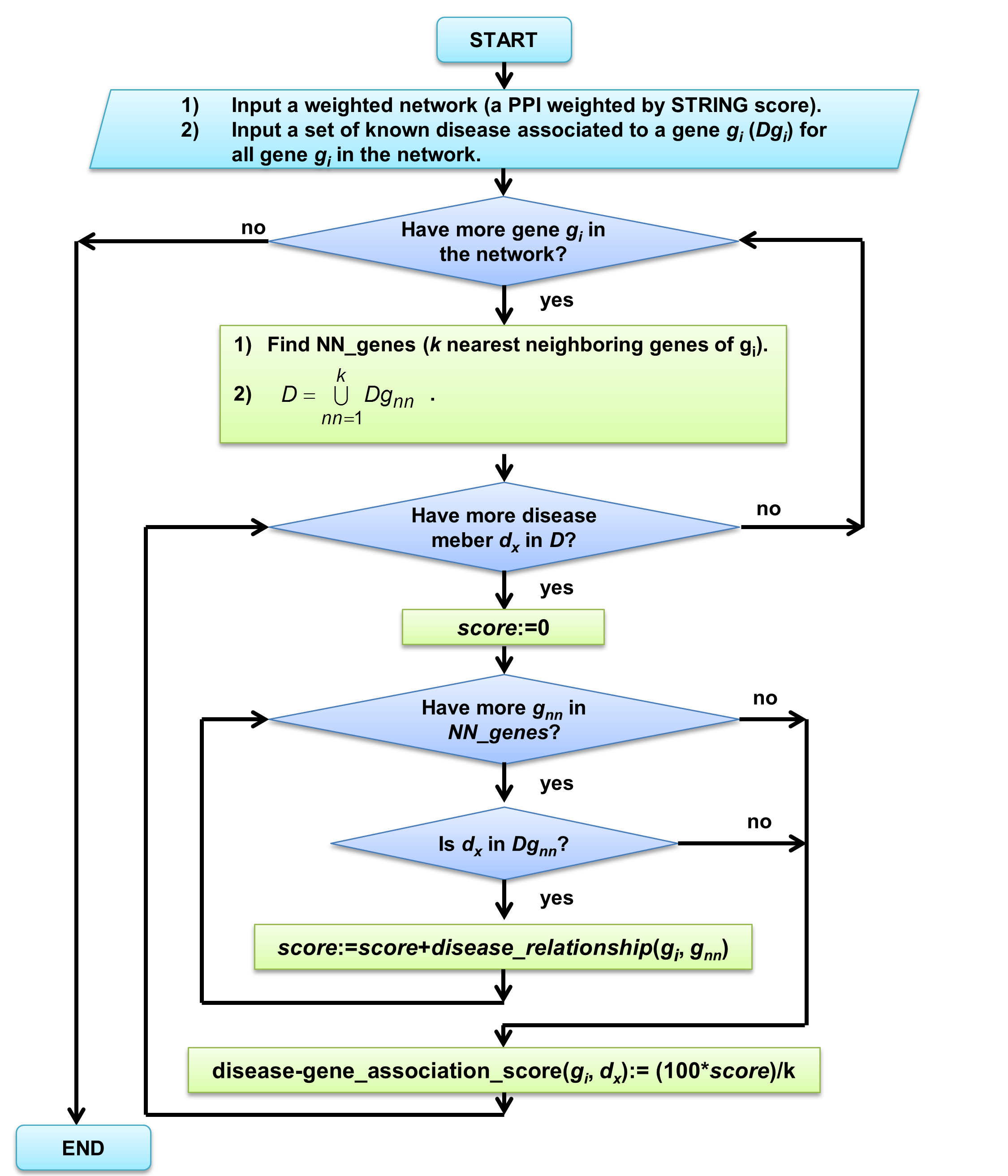

Supplement: S1 Fig — (TIF) [file pone.0199435.s006.tif]
